# Fatal Septic Meningitis in Child Caused by *Streptococcus suis* Serotype 24

**DOI:** 10.3201/eid2208.160452

**Published:** 2016-08

**Authors:** Anusak Kerdsin, Marcelo Gottschalk, Rujirat Hatrongjit, Shigeyuki Hamada, Yukihiro Akeda, Kazunori Oishi

**Affiliations:** Kasetsart University Chalermphrakiat Sakon Nakhon Province Campus, Sakon Nakhon, Thailand (A. Kerdsin, R. Hatrongjit);; University of Montreal, Montreal, Quebec, Canada (M. Gottschalk);; Osaka University, Osaka, Japan (S. Hamada, Y. Akeda);; National Institute of Infectious Diseases, Tokyo, Japan (K. Oishi)

**Keywords:** Streptococcus suis, serotype, meningitis, bacteria, bacterial infection, Thailand, streptococci

**To the Editor:** *Streptococcus suis* is a zoonotic bacterium that causes invasive infections in humans and pigs (*1*). Of the 29 described serotypes, serotype 2 is the most prevalent in humans, almost exclusively affecting adults (*1*). Other serotypes occurring sporadically in humans have been reported (*1*). Here we report a rare case of *S. suis* serotype 24 infection in a child.

A 2-year-old girl with Down syndrome was admitted to a hospital in Rayong Province, eastern Thailand, in May 2015. She had a high fever of 3 days’ duration, vomiting, stiff neck, rash, and purpura on her right leg and hip. The initial diagnosis was cellulitis and suspected meningococcal meningitis. Physical examination revealed a temperature of 39.5°C, pulse rate of 160 beats/min, respiratory rate of 80 breaths/min, and blood pressure of 94/55 mm Hg. Oxygen saturation was 80%, which is indicative of severe respiratory failure. An analysis of the complete blood count showed a leukocyte count of 21,460 cells/mL (83% neutrophils, 13% lymphocytes, 1% eosinophils, 3% monocytes) and platelet count of 155,000 cells/μL. A comprehensive metabolic panel test was not performed.

Bacteria were isolated from the patient's cerebrospinal fluid culture; however, hemoculture did not show any growth. Traditional biochemical tests and an API20Strep system assay (BioMérieux, Marcy l’Etoile, France) suggested that the organism was *S. suis*. The samples tested positive for *S. suis* serotype 24 by multiplex PCR and coagglutination testing (*2*,*3*). On the basis of these results, the condition was diagnosed as septic meningitis. Unfortunately, the patient died the day after admission, even though she had been treated with ceftriaxone on the day of admission.

The isolate from the child was susceptible to penicillin (MIC <0.12 μg/mL), ceftriaxone (MIC <1 μg/mL), erythromycin (MIC <0.25 μg/mL), levofloxacin (MIC <2.0 μg/mL), clindamycin (MIC <0.25 μg/mL), and vancomycin (MIC <1.0 μg/mL). Because breakpoints for *S. suis* are not defined in the 2015 Clinical and Laboratory Standards Institute guidelines, breakpoints for viridans streptococci were used instead (*4*). Multilocus sequence typing determined that the isolate was sequence type 221, which belongs to clonal complex (CC) 221/234. The previously described virulence markers (*epf, mrp,* and *sly* genes) were absent (*1*). These markers are mostly present in serotype 2 strains from Europe and Asia (*1*). We have shown that CC221/234 is a newly emerging, human infectious clone that includes serotypes 24 and 31, but not serotype 2, strains (*5*,*6*). Therefore, the presence of this CC should be monitored, and characterization of virulence factors of strains belonging to this CC should be further investigated.

The routes of *S. suis* infection include occupational exposure, recent contact with pigs or raw pork products, and recent consumption of raw pork products (*1*). This patient had no history of contact with pigs or pork products nor consumption of raw pig’s blood soup or any other source of undercooked meals before the onset of illness. *S. suis* may affect various other animal species (*7*), but the patient did not have any close contact with other animal species. Close family members of the patient did not report having consumed raw pork products, although they did report having close contact with pork meat for cooking. However, so far, no human cases have been confirmed to be the consequence of close contact with or consumption of undercooked meat from animal species other than pigs. In addition, human-to-human transmission of *S. suis* has not been reported (*8*). As reported in other similar cases, the route of the infection could not be confirmed in this case. A previous case of *S. suis* infection in a child was reported in a 1-month-old girl with meningitis in Thailand; however, certain details of that case, including the causative bacterial serotype, were not reported (Table) (*9*).

**Table:** Demographic and clinical characteristics for the 2 known reported *Streptococcus suis* infections in children

Characteristic	2015 case (this study)	2002 case (*9*)
Sex		
Age	2 y	1 mo
Temperature, °C	39.5	38.0
Pulse rate, beats/min	160	No data
Respiratory rate, breaths/min	80	No data
Blood pressure, mm Hg	94/55	No data
Leukocyte count, cells/mL	21,460	12,000
Diagnosis	Septic meningitis	Meningitis
Hearing loss	No data	No
Underlying disease	Down syndrome	No data
Outcome	Died	Survived
*S. suis* serotype	24	No data
Sequence type	221	No data

Although the isolation rate for *S. suis* serotype 24 strains remains low, increased awareness among clinicians treating patients with predisposing conditions is required given the emergence of *S. suis* infections caused by uncommon serotypes. Such awareness will be important for development of enhanced surveillance, epidemiologic control, and prevention strategies for public health.
